# Progress of Muscle Chain Theory in Shoulder Pain Rehabilitation: Potential Ideas for Pulmonary Rehabilitation

**DOI:** 10.1155/2022/2537957

**Published:** 2022-09-06

**Authors:** Shi Lv, Qian Wang, Qingbin Ni, Chunhua Qi, Yihong Ma, Simin Li, Yuzhen Xu

**Affiliations:** ^1^Department of Rehabilitation, The Second Affiliated Hospital of Shandong First Medical University, Taian 271000, China; ^2^Postdoctoral Workstation, Department of Central Laboratory, The Affiliated Taian City Central Hospital of Qingdao University, Taian 271000, China; ^3^Department of Neurology, Graduate School of Medical Sciences, Kumamoto University, Kumamoto 860-0811, Japan; ^4^Stomatological Hospital, Southern Medical University, Guangzhou 510280, China

## Abstract

Pulmonary dysfunction is very common in stroke patients. A study has shown that acute stroke patients often cause a series of pulmonary dysfunction due to primary damage to the respiratory center, which is an important reason for hindering disease treatment and recovery. American Thoracic Society (ATS) and the European Respiratory Society (ERS) pointed out that pulmonary rehabilitation (PR) can be applied to the rehabilitation of stroke patients to improve their lung function. PR can improve the respiratory muscle strength of stroke patients, which is beneficial to improving the respiratory function of patients. At the same time, it can also significantly increase the maximum oxygen intake of patients, effectively improve the cardiopulmonary function of stroke patients, and reduce respiratory complications such as aspiration pneumonia. However, the common dysfunction of joints and muscles such as shoulder pain after stroke will affect the process of pulmonary rehabilitation. This is mainly because the changes in the position of the shoulder girdle, the decrease in the range of motion of the cervical and thoracic spine, and the changes in the cervical spondylolisthesis position caused by the elevation of the upper limbs will directly affect the breathing movement during the pulmonary rehabilitation process. The instability of the spine will weaken the deep abdominal muscles and reduce the function of the diaphragm; moreover, changes in the alignment and stability of the cervical and thoracic spine will also lead to wrong breathing methods. Therefore, it is of practical clinical significance to evaluate the functional rehabilitation of shoulder joint muscles and evaluate the efficacy of stroke patients to improve their respiratory function. This article through an extensive review of domestic and foreign literature in recent years, combined with clinical practice experience, summarizes the practical application of chain structure theory in the fields of rehabilitation training, postural adjustment, pain relief, etc., and further studies the functional exercise method based on muscle chain theory. The research on the muscle chain of shoulder pain rehabilitation as a model illustrates the positive effect of reconstructing neuroarticular muscle function on the respiratory system, hoping to provide new ideas for the treatment of respiratory diseases in stroke patients.

## 1. Introduction

In 2019, the Global Burden of Disease Research Center showed that stroke has become the second leading cause of death in the world and the first leading cause of disability [1]. The incidence of stroke remains high due to the growth and aging of the world's population. Although the survival rate of the disease has improved, the remaining functional impairment still brings a great burden to the patient's family and society [2]. In the past, stroke rehabilitation mainly focused on physical movement, speech, and cognitive impairment, while the rehabilitation of pulmonary function has not been paid attention to and promoted. The reason may be that these patients did not show obvious pulmonary symptoms or diseases. But in fact, pulmonary dysfunction is very common in stroke patients [3]. A study [4] has shown that acute stroke patients often cause a series of pulmonary dysfunction due to primary damage to the respiratory center, which is an important reason for hindering disease treatment and recovery. This is mainly because the stroke will directly damage the corresponding respiratory center, form secondary nerve fiber damage around the lesion, destroy the nerve fiber motor conduction pathway and other pathways, damage the lung function of the patient, reduce the central respiratory driving force, and cause infection, cardiac. It is more likely to cause respiratory failure under stress conditions such as aging, which endangers the patient's life [5]. Therefore, the recovery of pulmonary function in stroke patients is a problem that cannot be ignored in the rehabilitation process. In recent years, pulmonary rehabilitation training has been increasingly used in the rehabilitation plan of stroke patients, mainly including aerobic exercise training, breathing training, and neuromuscular electrical stimulation (NMES). A study [6] has shown that pulmonary rehabilitation (PR) can improve the respiratory muscle strength of stroke patients, which is beneficial to improving the respiratory function of patients. At the same time, it can also significantly increase the maximum oxygen intake of patients, effectively improve the cardiopulmonary function of stroke patients, and reduce respiratory complications such as aspiration pneumonia. But for stroke patients, the presence of shoulder pain will greatly affect respiratory function (shoulder pain is one of the common complications of a stroke, with an incidence of 9%–40% [7]). In order to reduce the impact of shoulder pain, stroke patients often take a defensive posture, which makes the muscles around the shoulder appear tense and shortened, and the scapula is pulled forward. Due to the imbalance of the muscles around the shoulder and the change in the position of the scapula, there is a decrease in the upward rotation of the scapula and a decrease in the activity of the connection between the lower cervical spine and the upper thoracic spine. The reduced rotation of the scapula will restrict the important role of the scapulohumeral rhythm in shoulder joint activity and weaken the mechanical properties of the tendon. At the same time, the reduced mobility of the thoracic spine and scapula due to pain may increase the compensation of lumbar spine activity, thereby increasing the incidence of lumbar spondylosis [8]. Therefore, for stroke patients, changes in the position of the shoulder girdle, decreased cervical and thoracic spine activity, and changes in the posture of the cervical spondylolisthesis induced by lifting the upper limbs, auxiliary respiratory muscles (including the sternocleidomastoid, trapezius, pectoralis major), the continuous tension, etc. will directly affect the breathing movement [9]. The instability of the spine can weaken the deep abdominal muscles and reduce diaphragm function [10]. Moreover, changes in cervical and thoracic spine alignment and reduced stability can also lead to wrong breathing methods. Breathing movement is closely related to postural control and limb movement [11]. Therefore, it is of practical clinical significance to evaluate the functional rehabilitation of shoulder joint muscles and evaluate the efficacy of stroke patients to improve their respiratory function. The authors searched existing databases and found that there is very little research literature on this type of research, and there is a lack of objective clinical quantitative evidence for the impact of joint and muscle dysfunction on the respiratory system. This may be because most of the current clinical diagnoses, classifications, and treatments of shoulder pain are based on pathological anatomical diagnostic models [12]. Although this modality may aid in surgical decision-making, it is not necessarily effective in guiding patient rehabilitation. The inconsistent relationship between structural pathology and injury manifestations of shoulder pain makes it impossible to make treatment decisions based on pathological manifestations alone in clinical rehabilitation [13]. There are multiple studies [14, 15] that can be confirmed. In the asymptomatic population, there may also be structural damage to the shoulder tissue, and the degree of shoulder pain and functional limitation is poorly correlated with the degree and correlation of the structural defect on imaging. These studies raise questions about relying solely on imaging findings to determine the cause and treatment of shoulder pain. The pathological mechanism of shoulder pain is relatively complex, among which rotator cuff muscle injury is the most common cause of shoulder pain in the clinic. Fatigue and decreased contractility of shoulder muscles will directly affect the stability of shoulder structure and neuromuscular function control disorder. Further, there is an exacerbation of pain and functional limitations [16]. This requires medical personnel to study this problem from a new perspective. In recent years, muscle chain theory has been used more and more in the fields of rehabilitation training, posture adjustment, pain relief, and movement pattern improvement. A part of the body is not moved by a single muscle. Joints are controlled by several muscle pairs. These muscles join together into chains that work together to produce fluid movements. The muscle chain is the basis for ensuring the energy supply of the human kinematic chain system and cooperates with and influences the skeletal system and the nervous system [17]. In recent years, many researchers have proposed and established different muscle chain models from different angles. Zullo et al. [18] proved the continuity of muscle and fascia tissue from the physiological anatomy, which has a certain impact on the functional activities of the human body. Ellenbecker [19] also proposed that different muscle chains have an impact on human movement patterns. American Thoracic Society (ATS) and the European Respiratory Society (ERS) pointed out that PR can be applied to the rehabilitation of stroke patients to improve their lung function, but its specific application measures and effects have not been reached in the stroke rehabilitation guidelines consensus [20]. This article will use the theoretical research on the muscle chain of shoulder pain rehabilitation as a model to illustrate the positive effect of reconstructing neuroarticular muscle function on the respiratory system, hoping to provide new ideas for the treatment of respiratory diseases in stroke patients.

## 2. Theoretical Basis and Research Status of Muscle Chain

In the 1930s, Hans first introduced the concept of “link” into the human body when he studied muscles and explained that the kinematic chain in the body is the interconnection of each part of the muscle by function as a unit, and its meaning is not limited to the sum of each part of the muscle, but it is a chord-like chain structure in which muscles are continuously distributed in the body and undertake certain functions [21]. In 1964, American rehabilitation specialists Herman Kabat and Margaret Knott began to initially apply the muscle chain theory to clinical treatment methods. On this basis, Denys clarified the meaning of the concept of muscle chain and developed its application range, establishing an epoch-making “muscle chain” model, making it an effective clinical treatment method in the field of rehabilitation [22]. Vladimir Janda summarizes the theoretical differences between the structural school and the functional school in the field of rehabilitation and proposes the concept of “chain reaction,” which further expands the connotation of the human chain reaction; that is, the muscle chain, joint chain, and neural chain together form the human kinematic chain system [23]. This theory unifies the function and structure of the kinematic chain system, explains many phenomena that affect each other such as nerves, muscles, and bones, and makes the theoretical system of the kinematic chain more complete.

The modern systematic muscle chain theory system mainly includes Italian fascia manipulation, Meyers' “anatomy train” theory, Richard's spiral muscle chain theory, and Vladimir Janda's chain reaction theory system. Italian Fascial Manipulation focuses on self-symptoms and systemic networks and treats musculoskeletal disorders by improving body structure and movement efficiency [24]. Meyers' “Anatomy Train” Theory [25] believes that although muscles can function independently, they always affect the continuity of the body as a whole through the fascial network; Richard's spiral muscle chain theory believes that there are two important chains in the human body: the vertical muscle chain and the spiral muscle chain, the vertical muscle chain is used for relaxation and stability, and the spiral muscle chain is used for movement stability. Janda's theoretical system believes that the muscle chain is the basis for ensuring the function of the human kinematic chain system. The muscle chain is composed of synergists, muscle slings, and myofascial chains, which cooperate with and influence bones and nerves [17]:

1. *Synergists.* They refer to a muscle, tissue, or muscle group that cooperates with another muscle or muscle group to complete physical activities during physical activity and is the foundation for the development of basic movement patterns [26], including submotor, stabilizer, and neutralizer muscles. For example, when the shoulder rotates, the rotator cuff muscles are activated, and at the same time, the rhomboids, serratus anterior, and trapezius must act as scapula stabilizers to ensure the stability of the rotator cuff attachment points. False rotator cuff weakness may be caused by instability of the periscapular muscles
2. *Muscle Slings.* The slings are symmetrically distributed in the torso of the human body and are another form of muscle chain [27]. The muscular slings are integral, spanning multiple joints and providing and stabilizing, including the deep annulus brevis, which stabilizes the spine, and the superficial flat muscles, which support the stability of the trunk. The kinematic chain integrated by the muscle slings can better control the posture of the trunk above the hip joint so that the force can be effectively transmitted from all links of the kinematic chain to the limbs [26]
3. *Myofascial Chains.* They mainly include front and back surface lines, spiral lines, arm lines, functional lines, and front deep lines, which play a certain role in the core stability and posture maintenance of the human body from different levels and orientations [25]. Myofascia is a layer of connective tissue wrapped around muscle tissue. It runs through the deep layers of the skin, muscles, and organs from superficial to deep and mainly protects internal organs and sports tissues. Myofascia can store elastic potential energy, increase muscle work efficiency, and delay fatigue [28]. At present, through the Rolf School and other research, the myofascial chain has been expanded to 10 categories, a total of 20, which are spread all over the human body like a network [29]. When solving problems such as joint injury or pain, the path of the myofascia can be used to determine the cause of the injury, pain, and compensation phenomenon. The rational development of the internal function of the muscle chain is conducive to the coordination of the internal and external systems of the muscles, which is conducive to the efficient work of the muscles, and ultimately a better functional state.

## 3. Practical Application of Muscle Chain Theory in Clinic

For a fully functional and well-coordinated muscle chain, different muscle groups can exert continuous, orderly, and synergistic force to ensure a reasonable transfer of muscle energy between muscles. When one link is overactive and another is weak, it puts more pressure on the rest of the body, which can quickly lead to dysfunction [30]. In addition, there are qualitative differences between the muscle tissue and tendon tissue in the muscle chain in terms of structural characteristics, innervation, and nutrient supply. Compared with muscle tissue, tendon tissue has higher strength, poorer elasticity, and less nutrient supply, resulting in tendons to external stress. Stimulus sensitivity is weaker. Muscle-tendon junctions and tendon-bone junctions themselves are weak chain structural units and the weakest link in the muscle chain structure. Long-term repeated stretching and injury can easily lead to tissue degeneration, inflammation, and calcification [31]. In the muscle chain structure, as long as any link in the functional and structural imbalance reaches a certain level, joint disorders may occur.

The correct trajectory of the humerus within the glenoid, known as the glenohumeral rhythm, is critical for the proper functioning of the glenohumeral joint in throwing motion. Shoulder instability occurs when the muscles around the shoulder are unbalanced, and the humeral head cannot be maintained in the central position during movement, resulting in symptomatic dislocation [32]. Once a patient suffers from shoulder instability, it will seriously affect the patient's own normal life and normal shoulder function, reduce exercise ability, and have long-term shoulder pain symptoms, which will reduce the patient's quality of life [33]. The state of muscle imbalance around the shoulder is characterized by overactivation of the upper trapezius (UT), while the lower trapezius (LT), middle trapezius (MT), and serratus anterior (SA) tend to be underactive. It is particularly important to pay attention to the balance of UT/LT, UT/MT, and UT/SA in treatment [34, 35].

## 4. Application of Functional Exercise Based on Muscle Chain Theory in Shoulder Pain Rehabilitation

Proprioceptive neuromuscular facilitation (PNF) and muscle energy technology (MET) are based on muscle chain theory to improve neuromusculoskeletal system function, and there are many related studies. There are few studies on muscle chain exercise and shoulder pain rehabilitation alone. At present, it mainly starts from the aspect of myofascia.

### 4.1. Myofascial Trigger Point (MTrP) Theory for Diagnosis and Treatment of Shoulder Pain

Researchers [36] studying chronic nontraumatic shoulder pain found a higher prevalence of active and latent MTrP in the muscle. MTrP refers to the local highly sensitive tender points contained in palpable tight muscle bands in skeletal muscles, which can cause referred pain, muscle dysfunction, and even sympathetic neuralgia when pressed [37]. MTrP is divided into “active” and “latent.” Active trigger points often cause pain when palpated, while latent trigger points are generally asymptomatic and do not cause pain when palpated. Although not as painful as active trigger points, latent trigger points can also cause joint dysfunction [38].

Palpation is the only method available for the clinical diagnosis of myofascial pain, so reliable MTrP palpation is a necessary prerequisite for the effective diagnosis of shoulder myofascial pain. In an observational study [39], researchers palpated a total of 12 myofascial trigger points on both sides of the shoulder. The results of the study showed that the most reliable diagnostic features of myofascial trigger points were referred pain and jumping phenomenon. Percentage of pairwise agreement (PA) of referred pain ≥70% (range 63%–93%), and jump phenomenon PA ≥ 70% (range 67%–77%). Palpation of the infraspinatus is the most appropriate location to test for the presence of MTrPs in the shoulder (PA = 69%–80%).

Although myofascial trigger points have a greater impact on the degree of shoulder pain, the existing literature on the intervention effects of shoulder pain, activity limitation, and other functional disorders caused by various diseases seldom involves the treatment of MTrP [40]. Hsieh et al. [41] used acupuncture MTrP in the infraspinatus muscle, and the contralateral untreated MTrP was used as a control to evaluate the improvement of shoulder pain by visual analog scale (VAS). It was found that both active and passive ranges of motion of shoulder internal rotation and the pressure pain threshold of MTrP on the treated side have increased, and the pain intensity of the treated shoulder has reduced after dry needling. Bron et al. [42] performed MTrPs compressions, passive muscle stretching, and intermittent relaxation therapy on subjects and instructed patients to complete ergonomic muscle stretching and relaxation exercises by themselves. Compared with subjects without any treatment, the results showed that, after 12 weeks, the shoulder disorder score and VAS score in the intervention group were significantly improved compared with those in the control group (*P* < 0.05). Yamany and Salim [43] use low-level laser therapy (LLLT) to treat shoulder MTrPs. LLLT is a relatively uncommon, noninvasive treatment for musculoskeletal pain, in which nonthermal laser irradiation is applied to sites of pain. Forty patients with MTrPs of shoulder pain were randomly assigned to the active laser group and placebo laser group. Pain intensity by visual analog scale (VAS), active shoulder flexion and abduction by electrogoniometer, and pain pressure threshold (PPT) of trigger points by electronic digital algometer were measured before and after 4-weeks of treatment. After treatment, all the outcome measurements had shown significant improvement. Day [44] uses fascia manipulation to treat patients with chronic shoulder pain. Fascial manipulation is often used for local deep massage in specific areas of the deep fascia. This study tracked and evaluated patients treated with fascial manipulation for 3 months. The VAS scores of pain after 3 cycles were compared, and the results showed that the patients' shoulder pain decreased by an average of 57% after the 3rd cycle. In a randomized single-blind trial [45], the researchers aimed to evaluate dry needling on 1 latent MTrP, in conjunction with 1 active MTrP, in the infraspinatus muscle of older adults with nonspecific shoulder pain. Twenty patients over 65 years of age with unilateral or bilateral nonspecific shoulder pain and at least 1 active and 1 latent MTrPs in the infraspinatus were randomized into an experimental group (10 cases) and a control group (10 cases). The experimental group received deep dry acupuncture for the most painful active and latent MTrPs in the infraspinatus, while the control group received only one deep dry acupuncture. The evaluation of the pain threshold (PPT) of latent MTrPs in the anterior deltoid and extensor carpi radialis muscle, the evaluation of NRS, and the evaluation of grip strength were performed before the experiment, immediately after the experiment, and 1 week after the experiment. The measurement results showed that the pain intensity and grip strength immediately after the intervention (*P* < 0.05) and the PPT of the extensor carpi radialis muscle in the experimental group (*P*=0.009) were significantly improved compared with those before the intervention. One week after the intervention, the experimental group (*P*=0.008) and the control group (*P*=0.02), the pain intensity, the PPT of the extensor carpi radialis muscle in the experimental group (*P*=0.001), and the grip strength in the control group (*P*=0.029) were significantly improved. Shoulder pain patients are often accompanied by shoulder muscle MTrPs. Therefore, MTrPs should be one of the primary evaluation factors in the diagnosis and treatment of shoulder pain.

### 4.2. Application of Proprioceptive Neuromuscular Facilitation (PNF) in Shoulder Pain Rehabilitation

Richter's point of view that the human muscle chain was originally put forward by the American rehabilitationist Herman Kabat, who first pioneered PNF therapy for the treatment of polio patients [46]. PNF therapy is based on the chain movement of muscles, through stimulation of muscle and joint proprioceptors. (It is mainly located in the skin, muscles, ligaments, and free nerve endings. It transmits sensory impulses into the posterior cord of the spinal cord through peripheral nerves and then into the sensory center of the cerebral cortex through the medial lemniscus and then outputs motor feedback through the motor center system to adjust actions [47] and adopts a spiral diagonal movement pattern to promote recovery movement.) PNF therapy is actually a kind of exercise therapy. By integrating weak muscles into a muscle chain, using special stimulation composed of hearing, vision, and touch to act on the muscle chain, making full use of the unique functions of the nerve and muscle system, and making weak muscles are well integrated into the movement form of the muscle chain and ultimately achieve the function of rebuilding, restoring, and developing weak muscle areas [31]. The movement mode of PNF is named according to the movement direction and the end position. The main modes of upper limbs include the following: (1) D1 (diagonal) flexion: flexion-adduction-external rotation; (2) D1 extension: extension-abduction-internal rotation; (3) D2 flexion: flexion-abduction-external rotation; (4) D2 extension: extension-adduction-internal rotation. In addition to the helical diagonal movement, there are several facilitation methods in PNF that can help to strengthen shoulder stability, such as rhythmic initiation, rhythmic stabilization, dynamic reversal, isotonic, repetitive contractions, hold-relaxation, and retraction-relaxation, and other treatment techniques. A study [48] showed that compared with the treatment effect of joint mobilization alone, PNF combined with joint mobilization was more effective in improving shoulder pain and mobility impairment in patients.

In recent years, scholars have successively improved the basic operation methods and techniques of PNF technology, enabling patients with joint disorders to obtain more comprehensive PNF technology treatment, promoting the development of theory and practice, and being widely used in the rehabilitation of various bone and joint diseases [22]. In a study by Balcı et al. [49], PNF was used for patients with a frozen shoulder for>3 months, and the results showed that a single intervention of 1 hour through diagonal movement combined with rhythmic activation, repeated contractions, and other treatment techniques can effectively reduce shoulder pain to improve shoulder function and range of motion. Oledzka [50] adopts diagonal movement combined with rhythmic stabilization, hold-relaxation, and other treatment techniques to treat patients with subacromial impingement syndrome with a duration of ≤3 months for 40 minutes. The joint range of motion and pain in the rotation were improved compared with those before treatment. Kim et al. [51] investigated the effects of proprioceptive neuromuscular facilitation techniques and simple exercise on subjective pain reduction and blood flow velocity in patients with supraspinatus tears and assessed muscle recovery. Results showed statistically significant differences in blood flow velocity, visual analog scale, and disability scores in the arms, shoulders, and hands after 12 weeks of proprioceptive neuromuscular facilitation and simple exercise. In addition, the difference between proprioceptive neuromuscular facilitation techniques and simple movements was statistically significant. A systematic review [52] investigated the effectiveness of PNF treatment techniques in reducing pain and disability and increasing range of motion (ROM) and function in capsulitis. Among the 10 included studies, nine showed that the PNF group is superior in decreasing pain and reducing disability, increasing ROM, and improving function. The meta-analysis also showed a significant effect size and that the PNF is superior to conventional physical therapy in decreasing pain, increasing external rotation, and abduction ROM. Although PNF technology can effectively improve the pain level and joint range of motion in patients with bone and joint injuries, there is no research on the strength, time, angle, and optimal course of stretch during the treatment process, and its standardized treatment plan is not clear. In the research, our medical staff can further explore the application of PNF technology in the rehabilitation of bone and joint diseases.

### 4.3. Application of Muscle Energy Technology (MET) in Shoulder Pain Rehabilitation

Fryer [53] believes that the MET technology was originally developed by orthopedic surgeon Fred Mitchell to treat muscle imbalances, muscle shortening, muscle weakness, muscle limitations, and joint pain. The therapist precisely controls the magnitude and direction of the force, and the patient resists the therapist's resistance through muscle contraction, which can stretch the tight muscles and fascia, reduce muscle rigidity, strengthen weak muscles, and relieve pain [54]. This section summarizes the clinical research of MET at home and abroad in recent years, lists the manipulation methods adopted for bone and joint diseases, and provides a theoretical basis for clinical application. MET's basic model [55, 56] includes the following. (1) Contraction-relaxation (CR) technique: the therapist moves the patient's joint passively to the resting position or the position where resistance occurs, immobilizes, and makes the patient resist the force exerted by the therapist for 5–10 s. This technique can relax hypertonic muscle strength and restore proprioception. (2) Postisometric relaxation (PIR) technique: the therapist stretches the target muscle until it causes pain or can feel soft tissue resistance, allowing the patient to resist. Under the therapist's resistance, the target muscle contracts isometrically for 5–10 seconds. This technique can lengthen shortened muscles and fascia and reduce muscle tension. (3) Reciprocal Inhibition (RI) technique: the therapist stretches the target muscle until the patient experiences pain or feels soft tissue resistance and allows the patient to actively contract the antagonism of the target muscle. At the same time, the therapist applies resistance for 5–10 s and repeats it 3–5 times. (4) Contraction-relaxation-antagonist muscle contraction (CRAC) technology: this technology combines PIR and RI, firstly performs PIR on the target muscle, and then induces the target antagonist muscle contractions. This technique has a good therapeutic effect on joint contractures and is often used in the treatment of patients with chronic pain and scar adhesion.

Several studies have shown that the use of MET technology in combination with other rehabilitation methods can significantly improve joint range of motion and reduce pain. Chang Jiang [57] believes that extracorporeal shock waves combined with muscle energy technology can effectively improve the functional movement of shoulder joints in patients with shoulder joint dysfunction. A total of 60 patients with shoulder dysfunction were randomly divided into the control group and the observation group, with 30 cases in each group. The control group received conventional rehabilitation therapy, while the observation group received extracorporeal shock wave therapy and MET training on the basis of conventional rehabilitation therapy. The shoulder joint function was evaluated before treatment, 1 month after treatment, and 3 months after treatment, respectively. The results showed that at 1 month and 3 months after treatment, the Neer and UCLA scores of the two groups of patients were higher than those before treatment (*P* < 0.05), and the Neer and UCLA scores of the observation group at 1 month and 3 months after treatment were higher than those of the control group (*P* < 0.05). Xiong et al. [58] randomly divided 86 patients with a frozen shoulder into a control group and a treatment group. The control group was treated with Mulligan dynamic joint mobilization alone, and the treatment group was treated with MET technology at the same time. Abduction, external rotation, extension, adduction, internal rotation joint range of motion scores, and SPADI scores were significantly improved compared with those before treatment and were better than those of the control group (*P* < 0.05). Yuan and Liu [59] proposed that traditional combined therapy cannot completely solve the muscle-level problems caused by limited shoulder movement caused by the frozen shoulder, so MET technology is needed to restore muscle function. A number of studies have shown that MET technology has broad application prospects in the clinic and has a good curative effect on various diseases of the musculoskeletal system. It can be used in combination with traditional Chinese medicine therapy, joint mobilization, and physical factor therapy. The disadvantage is that there are few literature studies on the use of MET technology alone in the existing database; most of the studies use MET as an adjunct to other therapies, and it is difficult to determine the role of MET in combination with other therapies specific role. This also reflects the limitations of this single-treatment approach [60]. Moreover, MET technology lacks quantitative standards, and there will be certain differences in treatment effects.

## 5. Summary

Pulmonary rehabilitation (PR) was defined by the American Thoracic Society (ATS) and the European Respiratory Society (ERS) in 2013: Pulmonary rehabilitation is an individualized and comprehensive intervention after a comprehensive assessment of a patient's condition, including but not limited to exercise training, health education, and behavioral changes. etc., which aim to improve the physical and psychological status of patients with chronic respiratory diseases and promote long-term adherence to health-promoting behaviors [20]. Numerous trials [61–63] have demonstrated substantial improvements in dyspnea, exercise capacity, and health-related quality of life in patients following structured exercise training in PR programs. Exercise training can improve and prevent dyspnea and muscle dysfunction caused by airflow limitation and respiratory muscle and skeletal muscle dysfunction in patients with chronic respiratory disease, improve muscle function and exercise tolerance, reduce disease symptoms and disability, and delay disease. The process is the cornerstone of the PR program [64]. However, the current medical research on PR focuses on chronic obstructive pulmonary disease (COPD), and the observation indicators are mostly concentrated on respiratory function and quality of life. Representative literature includes research studies by Wootton et al. [65] and Cameron et al. [66]. However, there is a lack of objective clinical research on the effects of nerve, joint, and muscle dysfunction on the respiratory system in stroke patients. Parshall pointed out that the progress of dyspnea treatment does not match the research progress of the underlying mechanism, and more interdisciplinary translational research is needed to bridge the gap between the clinical treatment mechanism of dyspnea and the validation of dyspnea measurement [67]. Therefore, from the perspective of the motor system, this paper expounds on the positive effect of reconstructing neuroarticular muscle function on the respiratory system. The human kinematic chain is a macroscopic and abstract generalization of the three major chain structures of joint chain, muscle chain, and nerve chain. The joint chain includes the posture chain of all aspects of the body when the human body maintains a certain posture, the dynamic chain based on the spine, pelvis and scapula, sacroiliac joints, and the open chain and closed chain as the basic working form. Muscle chains are divided into three types: synergists, muscle slings, and myofascial chains, which mainly transmit tension, store elastic potential energy, compensate for movement, and maintain body stability. The neural chain is embodied in the sensory-motor system and neural development of motor patterns, which govern human movement and play a leading role in the formation and development of action patterns. The dynamic chain with complete structure and function not only ensures the reasonable transfer of muscle energy between muscles but also coordinates the synchronization, efficiency, order, and synergy of related tissues, ligaments, or muscle groups to varying degrees. The decline of the function of the kinematic chain link of a certain part of the body will lead to the decline of the adjacent related tissue structure and function and eventually lead to sports injuries [30]. Gambetta [68] proposed that when the human body performs the whole-body exercise, how to make multiple muscle groups and multiple joints cooperate to form a kinematic chain that conforms to the laws of biomechanics is a common problem for most sports training. In recent years, research theories on the deconstruction and integration of complex systems of the body have emerged in the field of sports human science, especially the cognition of functional training based on “chain structure” which has gradually become comprehensive and in-depth. Functional training is the result of abandoning and reflecting on the outdated ideas of traditional training methods such as simplicity, isolation, and mechanics. Functional training should be based on the scientific principle of the human body's chain structure as the theoretical basis and material basis, finding out the transmission mechanism of the chain reaction, which is of great significance for correcting the imbalance of body functions and sports injuries [26]. However, there are still some controversies about the medical issues of the chain structure, such as the central position of the human muscle chain, the priority of structural stability, and functional stability. The wave effect of the chain structure refers to the three-dimensional characteristics of the functional activity trajectory with an alternating sagittal plane and frontal plane, the surface muscles and deep muscles are fully mobilized, and the agonist and antagonist muscles work together in an orderly manner. In which direction you move, there are joints and muscles connected to it to maintain and support the body movements, so functional movements must pay attention to the synchronization and multidimensional effects of the relevant human kinematic chain reactions, so that the body can get multidimensional stimulation. For example, when performing waist core strength training, not only should the superficial erector spinae be exercised but also the deep muscles around the spine that maintain body stability and the iliopsoas on the frontal plane must also be paid attention to. Moreover, domestic and international rehabilitation training methods based on the chain structure have not yet formed a systematic system, and the operation methods also lack quantitative standards, resulting in differences in treatment effects. This paper only explores the impact on shoulder pain and dysfunction from the aspect of the muscle chain, so it also has certain limitations. In order to better promote the recovery of pulmonary function after stroke, we searched the database to summarize the advantages and applications of different types of functional exercise in pulmonary rehabilitation (Table 1), hoping to provide a reference for the treatment of respiratory diseases in stroke patients. In the future, our medical staff need to conduct rigorous clinical translational research on the design and evaluation of pulmonary rehabilitation in the fields of the nervous system and motor system, to explore the pathophysiological mechanisms affecting respiratory diseases and the impact of exercise rehabilitation on lung function. At the same time, we continue to conduct more in-depth exploration of early pulmonary rehabilitation in stroke patients.

## Figures and Tables

**Table 1:** Effects of different types of functional exercise on pulmonary rehabilitation.

Intervention index	Project	Method	Positive effects	Insufficient
Assessment of respiratory muscle strength helps to identify patients at risk for hypoventilation, to judge the effect of respiratory muscle training, and to assess respiratory muscle weakness and its severity [69], maximal inspiratory pressure (MIP), and maximal expiratory pressure (MEP) ≤80 cm H_2_O were defined as indicators of interventional therapy for respiratory muscle weakness [70]	Breathing training	Pursed lip breathing, abdominal breathing, stacked breathing, etc.	Breathing training can effectively promote alveolar expansion, increase body ventilation, and effectively improve lung function	Lack of more high-quality clinical randomized controlled trials to demonstrate the clinical significance of breathing training in improving lung function in stroke patients [71]
	Respiratory muscle training	Inspiratory muscle training, expiratory muscle training, combined inspiratory muscle-expiratory muscle training	Respiratory muscle training can significantly improve lung function and respiratory muscle strength and can reduce the incidence of respiratory diseases within 1 year after stroke [72]	How to make the best combination of different training methods and how to formulate the prescription of related training equipment still needs further research
	Airway clearance Techniques (ACT)	Active cycle of breathing techniques, percussion, postural drainage, cough training	ACT technology has a positive effect on improving the airway clearance rate and reducing pulmonary infection in stroke patients	ACT needs to be operated by professional therapists, but professional training is still in short supply, and its clinical promotion is still a problem to be solved in the short term
	Physical therapy techniques	Neuromuscular electrical stimulation, swallowing biofeedback stimulator, transcutaneous electrical nerve stimulation technology, etc.	Compared with traditional rehabilitation methods, the application of adjuvant physical therapy will greatly improve the efficiency of rehabilitation training for poststroke dysfunction	The indications of physical therapy techniques in stroke pulmonary dysfunction and the parameter selection of technical means still need a further clinical demonstration
	Exercise therapy	Aerobic exercise, joint mobilization, joint range of motion training, muscle strength training, etc.	Exercise training is the cornerstone of pulmonary rehabilitation, improving skeletal muscle strength, improving cardiovascular function, and thus improving patient mobility	The effects of exercise intensity, frequency, and other parameters on the pulmonary function of stroke still need to be further confirmed

## Data Availability

The data used to support the findings of this study are available from the corresponding author upon reasonable request.
